# Supplementary material for: NADH dehydrogenases Nuo and Nqr1 contribute to extracellular electron transfer by Shewanella oneidensis MR-1 in bioelectrochemical systems
Source: Sci Rep. 2019 Oct 18;9:14959. doi: 10.1038/s41598-019-51452-x (PMC6802487; doi:10.1038/s41598-019-51452-x)
Supplement: Supplementary file 1 — Supplementary Figures [file 41598_2019_51452_MOESM1_ESM.docx]

**NADH dehydrogenases Nuo and Nqr1 contribute to extracellular electron transfer by *Shewanella oneidensis* MR-1 in bioelectrochemical systems**

Cody S. Madsen^2^, and Michaela A. TerAvest^1*^

^1^Department of Biochemistry and Molecular Biology, Michigan State University, East Lansing, MI, USA

^2^Department of Biomedical Engineering, Michigan State University, East Lansing, MI, USA

*corresponding author

Address: 603 Wilson Rd., East Lansing, MI, 48823 Email: teraves2@msu.edu


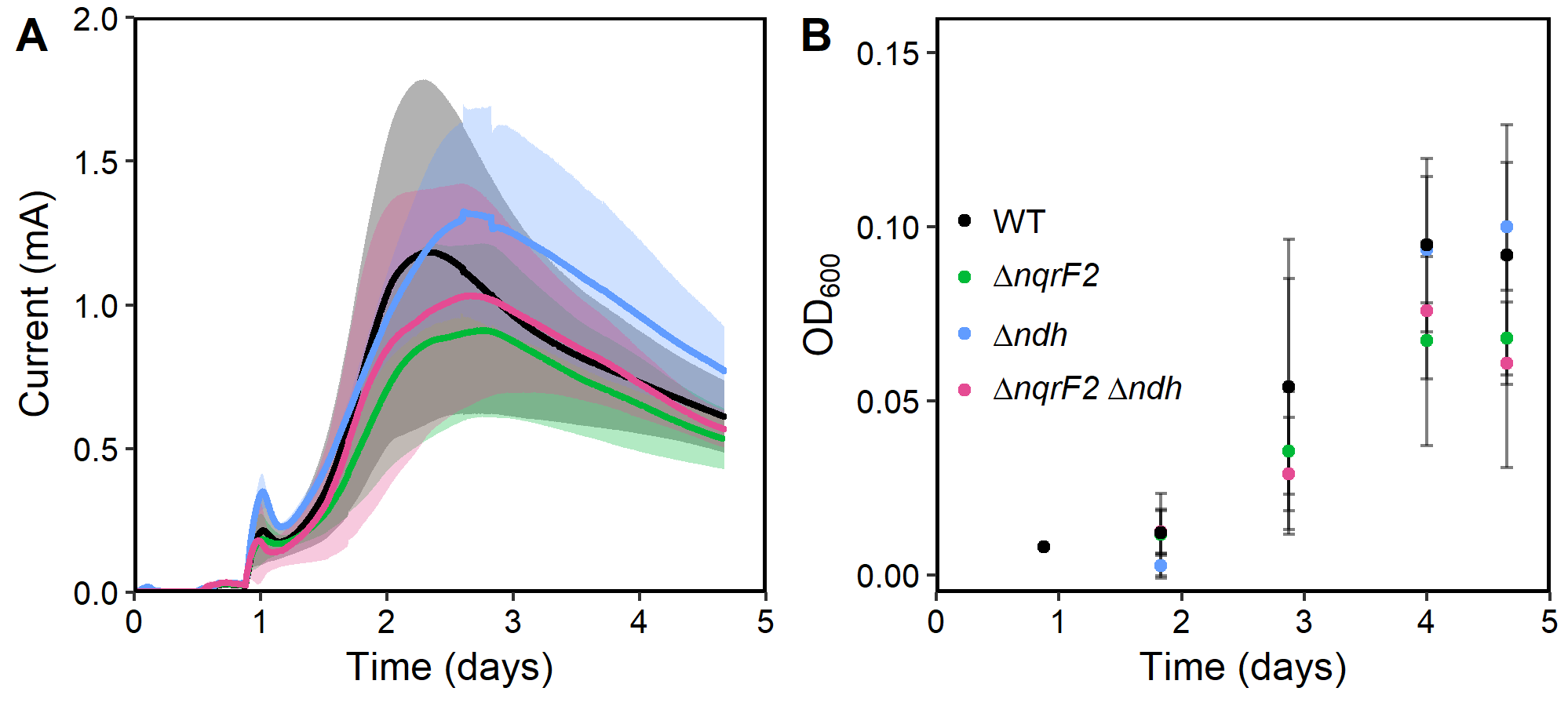


**Figure S1.** Current production (A) and OD_600_ (B) by WT, ∆*nqrF2*∆*ndh* and correlating single mutants with 10 mM NAG as the carbon source. Data analyzed in R with shaded regions and error bars indicating standard deviations (n=3).

*
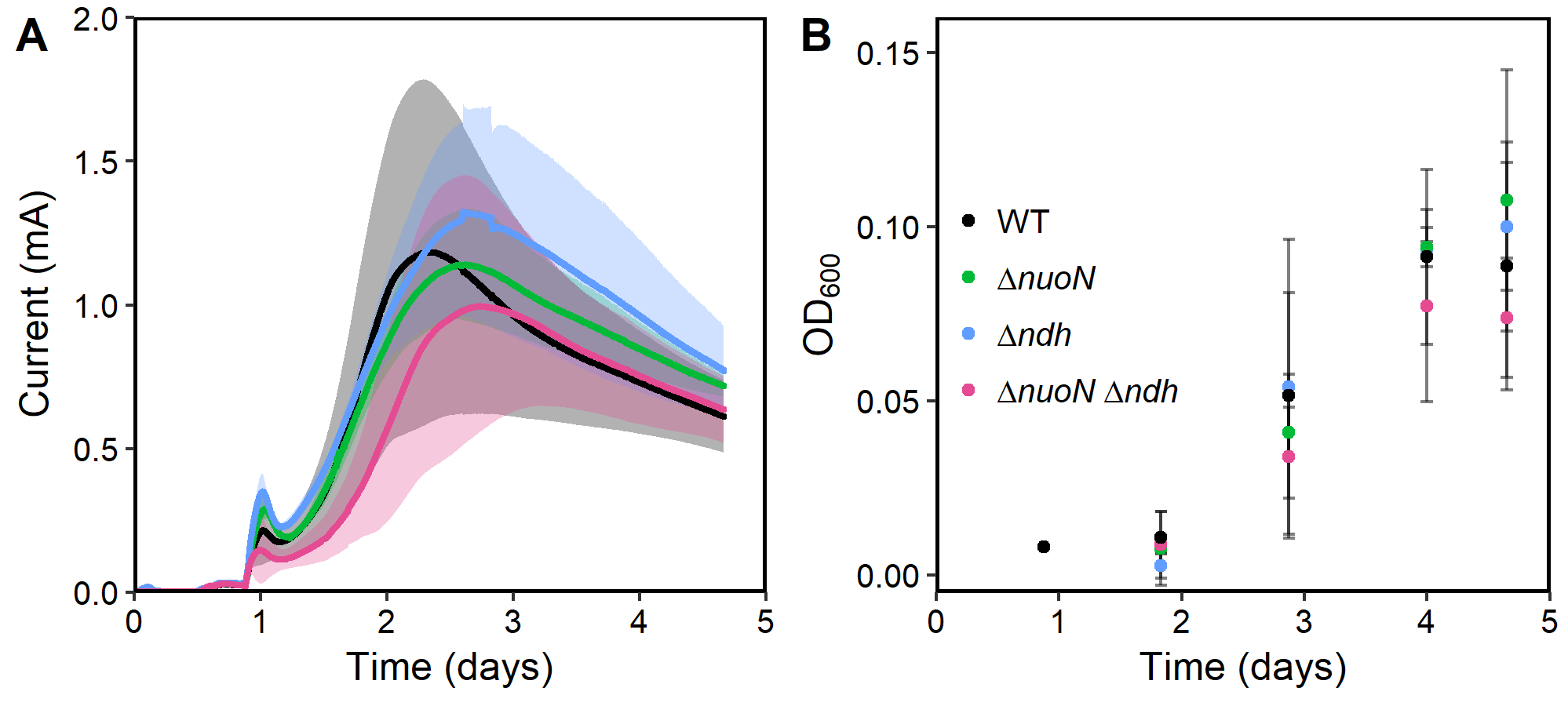
*

**Figure S2.** Current production (A) and OD_600_ (B) by WT, ∆*nuoN*∆*ndh* and correlating single mutants with 10 mM NAG as the carbon source. Data analyzed in R with shaded regions and error bars indicating standard deviations (n=3).


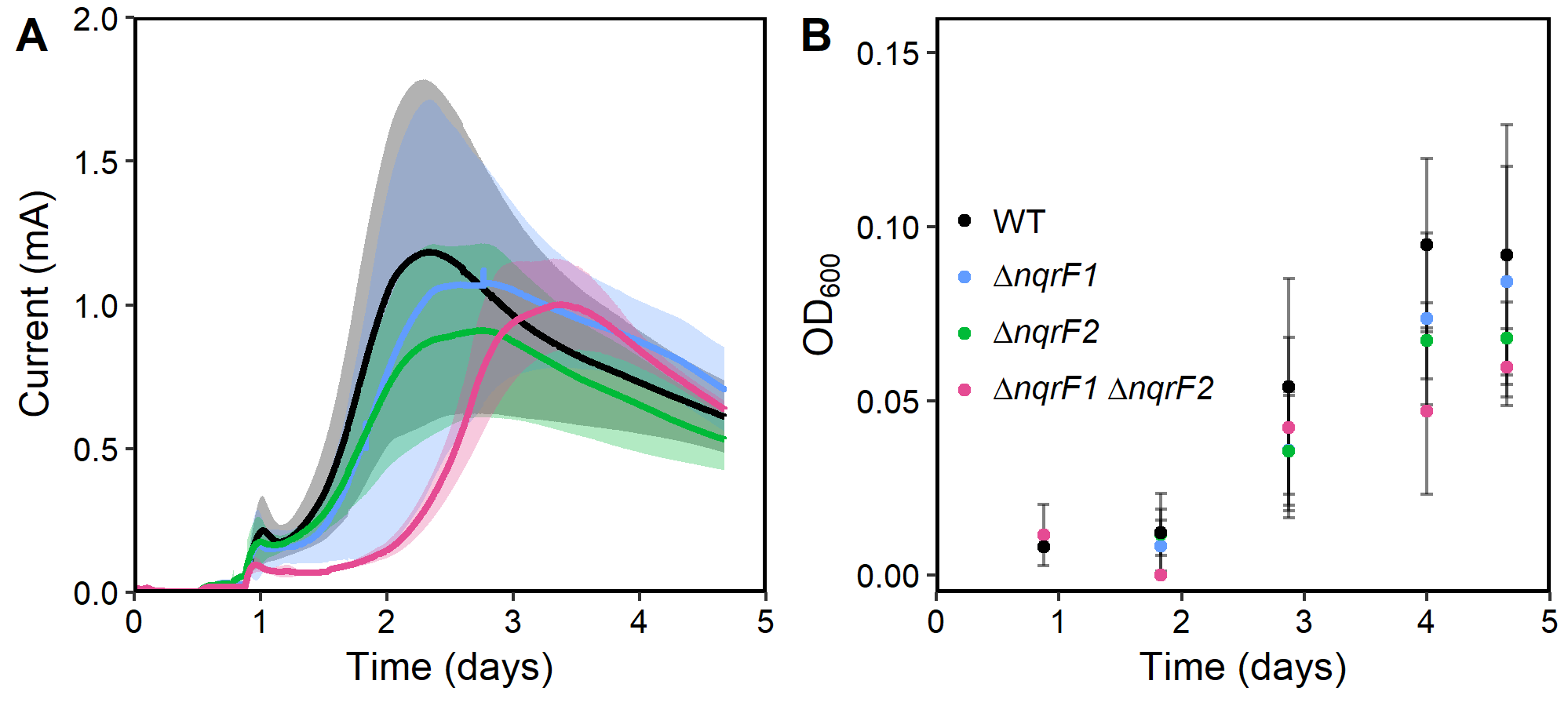


**Figure S3.** Current production (A) and OD_600_ (B) by WT, ∆*nqrF1*∆*nqrF2* and correlating single mutants with 10 mM NAG as the carbon source. Data analyzed in R with shaded regions and error bars indicating standard deviations (n=3).
